# Structural and functional roles of deamidation of N146 and/or truncation of NH_2_- or COOH-termini in human αB-crystallin

**Published:** 2011-09-14

**Authors:** C.O. Asomugha, R. Gupta, O.P. Srivastava

**Affiliations:** Department of Vision Sciences, University of Alabama at Birmingham, Birmingham, AL

## Abstract

**Purpose:**

The purpose of the study was to determine the relative effects of deamidation and/or truncation on the structural and functional properties of αB-crystallin.

**Methods:**

Using wild-type (WT) αB-crystallin and the αB deamidated mutant (i.e., αB N146D), we generated NH_2_-terminal domain deleted (residues no. 1–66; αB-NT), deamidated plus NH_2_-terminal domain deleted (αB N146D-NT), COOH-terminal extension deleted (residues no. 151–175; αB-CT), and deamidated plus COOH-terminal extension deleted (αB N146D-CT) mutants. All of the proteins were purified and their structural and functional (chaperone activity with insulin as target protein) properties were determined and compared to WT αB-crystallin.

**Results:**

The desired deletions in the αB-crystallin mutants were confirmed by DNA sequencing and matrix-assisted laser desorption/ionization time-of-flight (MALDI-TOF) mass spectrometric analysis. The homomers of αB-CT and its deamidated form (αB N146D-CT) became water insoluble, whereas the αB N146D, αB-NT, and αB N146D-NT species remained water-soluble. CD spectroscopic studies revealed that the mutants with deletion of NH_2_- or COOH-termini or deamidation showed increased β-sheet and decreased α-helical contents with the exception of αB N146D-CT, which showed a substantial increase in α-helix and decrease in β-sheet content. Results of intrinsic Trp fluorescence suggested little change in Trp microenvironment of αB N146D relative to WT αB, but substantial alterations on deletion of COOH-terminal extension or a combination of this deletion plus deamidation. Hydrophobic binding studies using the hydrophobic probe 8-anilino-1-naphthalene sulfonate (ANS) showed that, relative to WT αB structure, the N146 deamidation, COOH-terminal extension deletion or a combination of this deamidation and deletion resulted in a relatively compact structure whereas the NH_2_-terminal domain deletion and a combination of this deletion plus deamidation resulted in a relaxed structure. All the αB mutants showed higher molecular mass ranging between 1.2×10^6^ to 5.4×10^6^ Da, relative to WT αB which had a molecular mass of 5.8×10^5^ Da. Chaperone activity across all αB species decreased in the following order: WTαB > αB N146D-CT > αB N146D-NT > αB-NT > αB-CT > αB N146D. Specifically, substantial losses in chaperone activity (only 10% to 20% protection) were seen in αB N146D, αB-NT, and αB-CT. However, in the species with the combination of deamidation plus NH_2_- or COOH-terminal deletion, the percent protection was about 24% in αB N146D-NT and about 40% in αB N146D-CT.

**Conclusions:**

Although all mutants formed oligomers even after deamidation, on deletion of either NH_2_-terminal domain or COOH-terminal extension or a combination of these deletions and deamidation, their structural properties were substantially altered. The results suggested that the NH_2_-terminal domain is relatively more important than the COOH-terminal extension for the chaperone function of αB. The non-deamidated N146 residue, NH_2_-terminal domain and COOH-terminal extension are also of critical importance to the maintenance of αB-crystallin chaperone activity.

## Introduction

The α-, β-, and γ-crystallins are the major components of the vertebrate eye lens and their interactions lead to maintenance of the refractive properties of the lens, as well as lens transparency. Of the crystallins, α-crystallin accounts for almost half of the total lens protein and exists in vivo as an ~800 kDa hetero-oligomer made up of αA- and αB-crystallin in a 3:1 ratio [1,2]. Unlike αA-, αB-crystallin is a stress-inducible small heat shock protein (sHSP) [2,3] found in several organs other than the lens, such as the heart, skeletal muscle, and kidney [4,5]. In the brain, αB-crystallin has also been associated with neurologic disorders such as Alzheimer [6] and Parkinson [7] disease. As a member of the sHSP family, α-crystallins also function as molecular chaperones [8] to protect proteins from physiologic stress and bind improperly folded proteins to prevent their aggregation [3,9]. This chaperone function is thought to be a critical part of the maintenance of lens transparency.

Crystallins are long-lived proteins that must survive the lifetime of the lens and as such undergo post-translational modifications (PTMs) with age and cataract development. PTMs identified in the human lens include, but are not limited to, methionine oxidation, disulfide bond formation, deamidation of Asn and Gln residues, truncation of NH_2_- and COOH-termini, and backbone cleavage [10-12]. These PTMs, and others, are believed to alter protein structure and conformation and, in turn, the functional properties of the crystallins. Previous reports have shown that removal of 56 residues of the NH_2_-terminal domain and 32–34 residues of the COOH-terminal extension of both αA- and αB-crystallin results in improper folding, diminished chaperone activity, and formation of trimers and tetramers [5,13,14]. In a protein pin array assay of human αB-crystallin, a hydrophobic NH_2_-terminal sequence (residue no. 41 to 58) and polar COOH-terminal sequence (residue no. 155 to 165) were identified as interactive regions during complex assembly [15]. Although the deletion mutants, ∆41–58 and ∆155–165, exhibited larger complexes and greater polydispersity than wild-type (WT) αB, the chaperone activity of the ∆41–58 mutant was at the same level as WT, whereas the ∆155–165 mutant showed limited or no chaperone activity and poor solubility [15]. The study concluded that both NH_2_- and COOH-terminal regions of αB-crystallin play important roles in its solubility, stability and chaperone function.

The non-enzymatic process of deamidation introduces a negative charge by replacing a neutral amide group with a carboxylic group, and has been reported as the most common PTM in several studies [16-21], leading to protein destabilization and changes in solubility. The existence of deamidated fragments of αB-crystallin with N146 deamidation has been shown in both normal and cataractous lenses [22]. We also found that deamidation of N101 in αA-crystallin caused more pronounced changes in structural and functional properties than deamidation of N123 [23]. Likewise, deamidation of N146 had more pronounced effects on structural and functional properties of αB-crystallin than deamidation of N78 [24]. Similar findings of altered structure and function were seen in site-directed mutation studies of αB-crystallin [25], mutation of a conserved Arg residue (R120G) in the α-crystallin domain of αB-crystallin [26], as well as a study highlighting a novel mutation (D140N) in αB associated with the development of autosomal dominant congenital lamellar cataract [27]. Despite such studies, the role of deamidation in cataractogenesis is still unclear.

It has been suggested that deamidation of both Gln and Asn residues in proteins may serve as molecular clocks for biologic events including protein turnover, development, and aging and provide a signal for degradation to regulate intracellular levels [28,29]. This may explain why deamidation has been found to be the most commonly occurring PTM, as stated above. Also, since both αA- and αB-crystallin are susceptible to NH_2_- and COOH-terminal degradation, and deamidation sites exist in or near these regions (i.e., N101 and N123 of αA-crystallin and N78 and N146 of αB-crystallin), deamidation may serve as signal for truncation of these termini. Additional studies have shown that truncation or mutation in the COOH-terminal extension of αB-crystallin causes myofibrillar myopathies [30]. This raised the question of what were the relative effects of deamidation and/or truncation on the structural and functional properties of αA- and αB-crystallins. Therefore, we recently compared both deamidation and truncation of αA-crystallin and found that N123 deamidation, as well as truncation of NH_2_- and COOH-termini, altered the protein structure and were detrimental to chaperone function [31]. However, such comparison has not been performed in αB-crystallin.

To expand on our previous study of αA-crystallin [31], in the present study we sought answers as to what are the relative effects of deamidation and/or truncation of the NH_2_-terminal domain or the COOH-terminal extension on the structural and functional properties of αB-crystallin. This question was important because both deamidation and truncation of αB-crystallin have been extensively described in the literature. Therefore, in the present study using WT αB-crystallin and the αB deamidated mutant (i.e., αB N146D), we generated NH_2_-terminal domain deleted (residues no. 1–66), deamidated plus NH_2_-terminal domain deleted, COOH-terminal extension deleted (residues no. 151–175), and deamidated plus COOH-terminal extension deleted mutants and compared the structural and functional properties of these mutants to WT αB-crystallin.

## Methods

### Materials

The restriction endonucleases BamHI and SacI, the molecular weight protein markers and DNA markers were purchased from Amersham Biosciences (Piscataway, NJ), Invitrogen (Carlsbad, CA), and Promega (Madison WI), respectively. The T7 promoter, T7 terminator and other primers used in the study were obtained from Sigma-Aldrich (St. Louis, MO). Anti-Histidine-tagged mouse monoclonal primary antibody and goat anti-mouse IgG (H^+^L) horseradish peroxidase-conjugated secondary antibody were obtained from Calbiochem-EMD Biosciences (La Jolla, CA) and Thermo Scientific (Rockford, IL), respectively. Unless otherwise stated, all other molecular biology-grade chemicals used in this study were purchased from Sigma or Fisher Scientific (Fair Lawn, NJ).

### Bacterial strains and plasmids

The *E.coli* One Shot® TOP 10 cells and BL21 (DE3) bacterial strains were obtained from Invitrogen, and used for propagation and expression, respectively. The human, wild-type (WT) αB-crystallin cDNA cloned on a plasmid pDIRECT was received from Dr. Mark Petrash, University of Colorado, Denver, CO. Cells were propagated in Luria broth, and recombinant bacteria were selected using ampicillin.

### Generation of deletion mutants

**Figure 1:**
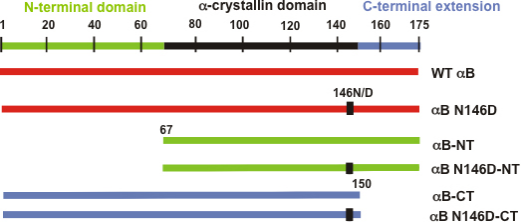
Schematic diagram showing the regions and residue numbers of the NH_2_-terminal domain, α-crystallin domain, COOH-terminal extension and deamidation sites in WT αB-crystallin and its mutants. WT αB-crystallin is a full-length protein containing all residues from 1 to 175. Residue N146 was deamidated (i.e., N to D) in the deamidated mutants. NH_2_-terminal domain deleted mutants (αB-NT and αB N146D-NT) are missing residues no. 1–66, while COOH-terminal extension deleted mutants (αB-CT and αB N146D-CT) are missing residues no. 151–175.

Deamidation of an Asn (N) residue at position 146 to an Asp (D) residue was introduced in αB-cDNA using the Quickchange site-directed mutagenesis kit (Stratagene, La Jolla, CA) as described previously [24]. The WT and deamidated αB cDNA were used as a template, along with specific complementary primer pairs (Table 1), to generate the desired deleted or deamidated plus deleted αB-crystallin mutants. The PCR products were ligated to a pET 100 Directional TOPO vector (Invitrogen). Recombinant human WT αB-crystallin and αB N146D were subcloned in the pET 100D TOPO vector to introduce a six His-tag at the NH_2_-terminus of the protein. The αB NH_2_-terminal domain (residue no. 1–66) or the COOH-terminal extension (residue no. 151–175) was deleted from the WT and the deamidated αB-crystallin mutant (i.e., αB N146D) using PCR-based deletion to generate NH_2_-terminal domain- or COOH-terminal extension-deleted mutants. The following four mutants (Figure 1) were generated: (i) αB-NT (NH_2_-terminally truncated [-NT]), (ii) αB N146D-NT, (iii) αB-CT (COOH-terminally truncated [-CT]), and (iv) αB N146D-CT. Briefly, 25 ng of template was used under the following PCR conditions: pre-denaturing at 95 °C for 30 s, followed by 30 cycles of denaturing at 95 °C for 30 s, annealing at 60–64 °C for 30 s (depending on the T_m_ of the primers), and extension/elongation at 72 °C for 1 min, with a final extension at 72 °C for 10 min. The PCR products were ligated to the pET 100 Directional TOPO vector (Invitrogen) as per the manufacturer’s instructions, and the positive clones were identified by restriction analysis using BamHI and SacI. The desired deletions were confirmed by DNA sequencing (Genomics Core Facility of the University of Alabama at Birmingham).

**Table 1:** Oligonucleotide primers used for subcloning WT αB-crystallin and deamidated αB species, and generating truncated αB mutant proteins using PCR-based deletion.

**Mutant constructs**	**Direction**	**Primers (5′–3′)**
WT αB	forward	CACCATGGACATCGCCATCCACCACCCCTG
	reverse	CTATTTCTTGGGGGCTGCGGTGACAGC
αB N146D	forward	CACCATGGACATCGCCATCCACCACCCCTG
	reverse	CTATTTCTTGGGGGCTGCGGTGACAGC
αB-NT	forward	CACCCGCCTGGAAAAGGACAGGTTCTCTG
	reverse	CTATTTCTTGGGGGCTGCGGTGACAGC
αBN146D-NT	forward	CACCCGCCTGGAAAAGGACAGGTTCTCTG
	reverse	CTATTTCTTGGGGGCTGCGGTGACAGC
αB-CT	forward	CACCATGGACATCGCCATCCACCACCCCTG
	reverse	TTATTTCCTTGGTCCATTCACAGTGAG
αBN146D-CT	forward	CACCATGGACATCGCCATCCACCACCCCTG
	reverse	TTATTTCCTTGGTCCATTCACAGTGAG

NT and CT denote the NH_2_-terminally truncated and COOH-terminally truncated mutant proteins, respectively.

### Expression and extraction of WT and mutant proteins in soluble and inclusion bodies

Positive PCR amplicons were transformed into *E. coli* BL21 (DE3) cells using a standard *E. coli* transformation technique, as previously described [23,24]. The proteins were overexpressed by addition of IPTG (Isopropyl β-D-1-thiogalactopyranoside; final concentration of 1 mM), and the cell cultures were incubated further at 37 °C for 4 h. The cells were harvested and resuspended in lysis buffer (25 mM Tris-HCl [pH 7.8], 50 mM NaCl, 0.9% glucose, 1 mM EDTA, containing lysozyme (0.25 mg/ml) and protease inhibitor cocktail [Sigma]) and sonicated while kept on ice. DNA was degraded by treatment with DNase I (10 μg/ml) for 30 min on ice. The soluble fraction was separated by centrifugation at 8000× g for 10 min at 4 °C, and the insoluble fraction was resuspended in a detergent buffer (DB; 0.5 M NaCl, 1% [w/v] sodium deoxycholate, 1% NP-40, and 20 mM Tris-HCl, pH 7.5). The detergent-soluble fraction was separated by centrifugation at 5000× g for 10 min at 4 °C. The resultant pellet was washed with 0.5% Triton X-100 and centrifuged as stated above. Washing of the pellet was repeated as necessary to remove bacterial debris from the inclusion bodies. The final pellet was resuspended in denaturating binding buffer (DBB; 8M urea, 0.5 M NaCl, and 20 mM sodium phosphate, pH 7.8).

### Purification of WT and mutant proteins

Depending on the expression of the desired mutant proteins in either soluble fractions or in inclusion bodies (insoluble fractions), each protein was purified under either native or denaturing conditions. In case the desired protein was expressed in a partly soluble form (i.e., present in both soluble fraction and inclusion bodies), the soluble protein fraction was selectively used for its purification. All purification steps, including refolding of proteins, were performed at 4 °C unless otherwise indicated. Each protein was purified by affinity chromatography using Invitrogen ProBond Ni^2+^-chelating columns according to the manufacture’s instructions. Briefly, under native conditions, the column was equilibrated and a protein preparation was applied to the column using a native binding (NB) buffer (20 mM sodium phosphate containing 0.5 M NaCl, pH 7.8), followed by washing with NB buffer containing 20 mM imidazole (pH 7.8) and elution of column-bound protein with NB containing 250 mM imidazole (pH 7.8). Under denaturing conditions, the column was equilibrated with DBB. Following the application of desired protein preparation, the unbound proteins were eluted by a first wash with DBB, followed by a second and third wash with DBB at pH 6.0 and pH 5.3, respectively. Finally, bound proteins were eluted with DBB containing 250 mM imidazole (pH 7.8).

SDS–PAGE analysis [32] was used to analyze the fractions recovered from Ni^2+^-affinity column chromatography that contained the desired protein after purification. Proteins purified under native conditions were dialyzed against 50 mM phosphate buffer (pH 7.8) at 4 °C, and stored at −20 °C until they were used. The proteins purified under denaturing conditions were refolded using a previously published method [33] as described below.

### Refolding of proteins purified under denaturating conditions

Proteins purified under denaturing conditions were refolded by dialysis for 24 h at 4 °C against 50 mM sodium phosphate (pH 7.5) containing 1 mM DTT and decreasing urea concentrations from 8 M to 4 M, and finally in a urea-free phosphate buffer.

### Characterization of structural/functional properties of WT αB-crystallin and Its deamidated and deamidated plus deleted mutants

#### Circular dichroism (CD) spectroscopy

To investigate the conformational changes in purified WT αB-crystallin and the N146D deamidated or deamidated plus deleted mutant proteins, their far-UV CD spectra were recorded at room temperature over a range of 195 – 260 nm on a Jasco J815 CD spectrometer (Jasco, Inc., Easton, MD) using 0.2 mg/ml of protein in 50 mM sodium phosphate buffer (pH 7.8), as previously described [31]. A quartz cell of 0.5 mm path length was used, and the reported spectra are the average of five scans, which were corrected for the buffer blank and smoothed. The secondary structural contents of WT and mutant proteins were determined using the SELCON3 analysis program.

#### Fluorescence studies

All fluorescence spectra were recorded in corrected spectrum mode using a Shimadzu RF-5301PC spectrofluorometer (Shimadzu Corporation, Columbia, MD) with excitation and emission bandpasses set at 5 and 3 nm, respectively. The intrinsic Trp fluorescence intensities of the WT αB-crystallin, the N146D deamidated mutant, and the deamidated plus COOH-terminal extension-deleted mutants (0.2 mg/ml of protein in 50 mM sodium phosphate buffer, pH 7.8) were recorded with excitation at 295 nm and emission between 300 and 400 nm. Because human αB-crystallin contains Trp residues at positions 9 and 60 that were lost during deletion of the NH_2_-terminal domain (residue no. 1–66), the total fluorescence intensities of the NH_2_-terminally deleted mutants (i.e., αB-NT and αB N146D-NT) were recorded with excitation at 290 nm and emission between 300 and 400 nm.

#### ANS binding and fluorescence spectroscopy

The binding of a hydrophobic probe, 8-anilino-1-naphthalene sulfonate (ANS), to WT αB-crystallin, deamidated, or deamidated plus NH_2_- or COOH-terminally deleted αB-crystallin mutants was determined by recording fluorescence emission spectra between 400 and 600 nm after excitation at 390 nm, as previously described [23,24]. In these experiments, 15 μl of 0.8 mM ANS (dissolved in methanol) was added to 0.2 mg/ml of protein dissolved in 50 mM phosphate buffer (pH 7.8), mixed thoroughly, and incubated at 37 °C for 15 min before spectroscopy.

#### Oligomer size determination by dynamic light scattering

A multiangle laser light scattering instrument (Wyatt Technology, Santa Barbara, CA) coupled to an HPLC system was used to determine the absolute molar mass of the WT protein and its mutant proteins. Prior to their analysis, protein samples in 50 mM sodium phosphate (pH 7.8) were filtered through a 0.22 μm filter. Results were acquired using 18 different angles, which were normalized with the 90°-angle detector.

#### Chaperone activity assay

Chaperone activity of homoaggregates of WT αB-crystallin, αB N146D, αB-NT, αB N146D-NT, αB-CT, and αB N146D-CT mutants was determined using methods previously described [24]. The aggregation of insulin by reduction with 20 mM DTT at 25 °C, either in absence or at a 1:1 ratio (insulin: αB-crystallin protein) of different αB-crystallin species, was determined. Aggregation was monitored using light scattering at 360 nm as a function of time using a Shimadzu UV-VIS scanning spectrophotometer (model UV2101 PC) equipped with a six-cell positioner and a temperature controller (Shimadzu model CPS-260).

## Results

### Confirmation of site-specific deletions in αB-crystallin mutants

WT αB-crystallin and a deamidated αB-crystallin mutant (i.e., αB N146D) previously generated in our laboratory [24], were used as templates to generate four NH_2_-terminal domain-deleted or COOH-terminal extension-deleted mutants (see Methods). The NH_2_-terminally deleted (-NT) and deamidated plus NH_2_-terminally deleted mutants are referred to in the text as αB-NT and αB N146D-NT, respectively, whereas the COOH-terminally deleted (-CT) and deamidated plus COOH-terminally deleted mutants are referred to in the text as αB-CT and αB N146D-CT, respectively. DNA sequencing results confirmed the desired deletions: αB-NT and αB N146D-NT (containing residues no. 67–175), and αB-CT and αB N146D-CT (containing residues no. 1–150).

### Expression and purification of WT αB-crystallin and mutant proteins

WT αB-crystallin and mutant protein expression was induced in the BL21 (DE3) expression cell line using 1 mM IPTG for 4 h, as previously described [24], and proteins were recovered in either the soluble fraction, insoluble fraction (inclusion bodies), or both fractions (Table 2). WT αB-crystallin, αB N146D, αB-NT, and αB N146D-NT proteins were recovered in the soluble fraction, whereas αB-CT and αB N146D-CT proteins were recovered in the insoluble fraction. These results were consistent with the known solubility properties of the NH_2_-terminal domain and COOH-terminal extension. Therefore, upon deletion of the hydrophobic NH_2_-terminus, the proteins remained soluble, however, on deletion of the hydrophilic COOH-terminus, the proteins were insoluble.

**Table 2:** Presence of WT αB-crystallin and its deamidated, NH_2_-terminal domain or COOH-terminal extension deleted, and deamidated plus deleted mutants in the soluble fraction or inclusion bodies.

**WT αB- and mutant crystallin species**	**Soluble fraction**	**Inclusion bodies**
WT αB	+	-
αB N146D	+	-
αB-NT	+	-
αB N146D-NT	+	-
αB-CT	-	+
αB N146D-CT	-	+

The + sign indicates presence of the protein in the soluble or insoluble fraction based on western blot and SDS–PAGE analyses.

**Figure 2:**
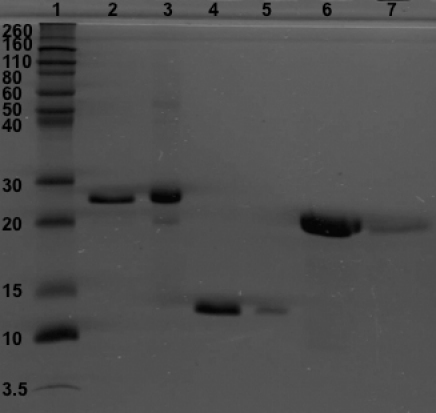
SDS–PAGE analysis of purified, His-tagged WT αB-crystallin and its deamidated, NH_2_- or COOH-terminally deleted mutants, and deamidated plus deleted mutants, following Ni^2+^-affinity column purification (see Methods). Lane 1 – molecular weight marker; Lane 2 – WT αB-crystallin; Lane 3 – αB N146D; Lane 4 – αB-NT; Lane 5 – αB N146D-NT; Lane 6 – αB-CT; αB N146D-CT.

Following overexpression of proteins in *E. coli* at 37 °C, each protein was purified to almost homogeneity using Ni^2+^-affinity columns under native or denaturing conditions (see Methods). On SDS–PAGE analysis, the purified His-tagged WT αB-crystallin and deamidated mutant proteins (containing residues 1–175) showed molecular weights (M_r_) of ~27 kDa (Figure 2, lane 2 and 3), whereas His-tagged NH_2_-terminally deleted (containing residues no. 67–175) and COOH-terminally deleted (containing residues no 1–150) species showed lower M_r_’s of ~15 and ~20 kDa, respectively (Figure 2, lanes 4–7). As seen by SDS–PAGE analysis, WT αB and its mutant proteins were recovered in highly purified forms and showed M_r_’s higher than expected due to the addition of six His residues.

### Comparison of properties of WT αB-crystallin, deamidated and deamidated plus deleted mutants

#### Circular dichroism spectral studies

**Figure 3:**
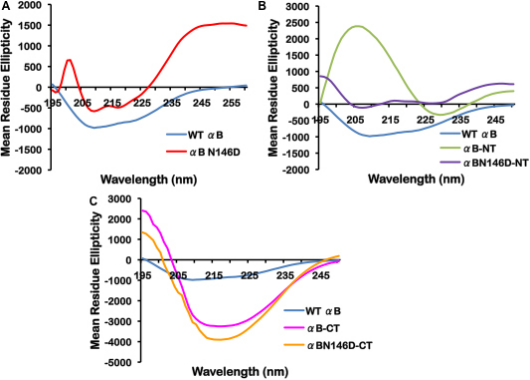
Far-UV CD spectra of WT αB-crystallin and its mutant proteins. Spectra were recorded using protein preparations of 0.2 mg/ml, dissolved in 50 mM sodium phosphate buffer (pH 7.8), and a cell path length of 0.5 mm. The reported spectra are the average of 5 scans corrected for the buffer blank and smoothed. **A**: WT αB-crystallin and deamidated mutant. **B**: WT αB-crystallin and NH_2_-terminal domain deleted mutants. **C**: WT αB-crystallin and COOH-terminal extension deleted mutants.

To evaluate the effects of deletion of the NH_2_-terminal domain or COOH-terminal extension on the secondary structure of WT αB-crystallin and a deamidated mutant protein, far-UV CD spectra and secondary structural content were determined (Figure 3, Table 3). As seen in Table 3, SELCON3 software analysis of secondary structural content showed that WT αB-crystallin contained predominantly β-sheet structure with 19.3% α-helix, 48.7% β-sheet, 12.4% β-turn, and 19.6% random coil. Conversely, the αB N146D mutant showed 6.1% α-helix, 64.2% β-sheet, 5.5% β-turn, and 24.2% random coil, suggesting that deamidation at N146 considerably altered α-helical and β-sheet content (Figure 3, Table 3). Likewise, deletion of either the NH_2_-terminal domain or COOH-terminal extension alone resulted in increased β-sheet content and decreased α-helical content relative to WT αB. αB-NT showed a greater reduction in α-helix (i.e., 3.0% α-helix, 69.7% β-sheet, 16.9% β-turn, and 10.6% random coil), while αB-CT showed a minimal reduction in α-helix (i.e., 11.7% α-helix, 62.2% β-sheet, 10.5% β-turn, and 17.1% random coil; Figure 3B,C, Table 3). However, both αB-NT and αB-CT showed similar increases in β-sheet content. With NH_2_-terminal domain deletion of the deamidated αB-crystallin, α-helical and β-sheet contents were similar to the content of the deamidated alone species (i.e., αB N146D-NT showed 5.9% α-helix, 62.5% β-sheet, 5.5% β-turn, and 24.4% random coil), suggesting that NH_2_-terminal truncation alone exhibits relatively greater structural changes than the N146 deamidation. On COOH-terminal extension deletion of the deamidated αB-crystallin, α-helical content substantially increased while β-content decreased relative to both WT and the deamidated alone species (i.e., 47.5% α-helix, 31.7% β-sheet, 6.8% β-turn, and 14.8% random coil). This suggested that the combination of N146 deamidation and COOH-terminal deletion had greater affect on secondary structural content than COOH-terminal deletion alone.

**Table 3:** Secondary structural content of WT αB-crystallin and its mutants.

**Crystallin Species**	**α Helix (±1%)**	**β Sheet (±1%)**	**β Turn (±1%)**	**Random coil (±1%)**
WT αB	19.3	48.7	12.4	19.6
αB N146D	6.1	64.2	5.5	24.2
αB-NT	3.0	69.7	16.9	10.6
αB N146D-NT	5.9	62.5	5.5	24.4
αB-CT	11.7	62.2	10.5	17.1
αB N146D-CT	47.5	31.7	6.8	14.8

Percentages were determined by analysis of the Far-UV spectra (Figure 3) using the SELCON3 analysis program.

#### Intrinsic Trp fluorescence and total fluorescence

**Figure 4:**
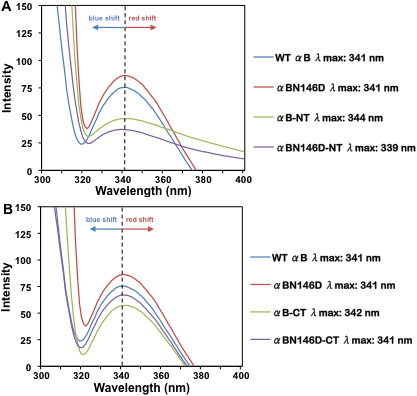
Intrinsic Trp and total fluorescence spectra of WT αB-crystallin and its mutant proteins. **A**: Total fluorescence spectra (Ex 290 nm, Em 300–400 nm) were recorded for the NH_2_-terminal domain deleted mutants because Trp residues 9 and 60 were deleted along with the deletion of this domain. **B**: Intrinsic Trp fluorescence spectra (Ex 295 nm, Em 300–400 nm) were recorded for mutants containing the NH_2_-terminal domain. The dotted lines indicate the wavelength of maximum peak fluorescence (λ_max_) observed in WT αB-crystallin, used to determine whether a blue or red shift in wavelength occurred.

The tertiary structure of a protein can be altered by changes in its secondary structure, therefore it is necessary to determine if such changes have occurred given the changes in secondary structural content observed above. One way to do this is by examining the alterations in fluorescence spectra of hydrophobic residues such as Trp. The NH_2_-terminal domain of WT αB-crystallin (residues no. 1–66) contains two Trp residues at positions 9 and 60. However, upon deletion of the NH_2_-terminal domain, these two residues were deleted. Therefore, the intrinsic Trp fluorescence spectra of species containing both Trp residues (i.e., WT αB-crystallin, αB N146D, αB-CT, and αB N146D-CT) and the total fluorescence spectra of species lacking the Trp residues (i.e., αB-NT and αB N146D-NT) were recorded from 300 – 400 nm with excitation at 295 nm and 290 nm, respectively (Figure 4A,B). On intrinsic Trp fluorescence, WT αB and the αB N146D species showed identical fluorescence with λ_max_ peak at 341 nm, though αB N146D emission intensity was slightly increased relative to WT αB (Figure 4A,B). On COOH-terminal extension deletion, both αB-CT and αB N146D-CT showed noticeably diminished fluorescence intensity compared to WT αB-crystallin, but minimal to no shift in λ_max_ with λ_max_ peaks at 342 nm and 341 nm, respectively (Figure 4B). This suggested that COOH-terminal extension deleted species showed almost no change in microenvironment around the Trp residues, but their Trp residues were relatively less exposed. On total fluorescence determined by excitation at 290 nm, αB-NT showed a 3 nm red shift with λ_max_ at 344 nm and a substantial decrease in fluorescence intensity; however αB N146D-NT showed a 2 nm blue shift with λ_max_ 339 nm and the maximum decrease in fluorescence intensity of all the species. These results suggest that NH_2_-terminal domain deletion also causes a change in microenvironment, with the most notable change occurring with the combination of both deamidation and NH_2_-terminal deletion. Taken together, the results suggest that NH_2_-terminal domain deletion caused greater changes in tertiary structure, compared to the COOH-terminal extension deletion or deamidation at N146 alone.

#### Surface hydrophobicity

**Figure 5:**
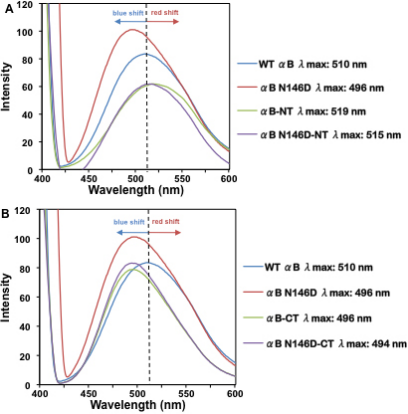
Fluorescence spectra of WT αB-crystallin and its mutants following ANS binding. Spectra were recorded by excitation at 390 nm and emission from 400 to 600 nm using 0.2 mg/ml protein preparations mixed with 15 μl of 0.8 mM ANS (dissolved in methanol) and incubated at 37 °C for 15 min. **A**: WT αB-crystallin and its deamidated and NH_2_-terminal domain deleted mutants. **B**: WT αB-crystallin and its deamidated and COOH-terminal extension deleted mutants. The dotted lines indicated the wavelength of maximum peak fluorescence (λ_max_) observed in WT αB-crystallin, used to determine whether a blue or a red shift in wavelength occurred.

In light of previous studies suggesting that binding of target proteins during chaperone function is associated with exposed hydrophobic surfaces of the chaperone molecule [34-36], we investigated exposed hydrophobic surfaces of the WT αB-crystallin and mutant proteins using a hydrophobic fluorescence probe, ANS (Figure 5). ANS is a useful surface hydrophobicity probe because it remains non-fluorescent in aqueous solutions until bound to hydrophobic surfaces, therefore its fluorescence correlates to its binding. On ANS binding, WT αB-crystallin exhibited fluorescence with λ_max_ peak at 510 nm (Figure 5A,B), but a relative 14 nm blue shift with increased fluorescence occurred on N146 deamidation. The NH_2_-terminal domain deleted mutant exhibited a 9 nm red shift with λ_max_ at 519 nm and decreased fluorescence intensity compared to WT (Figure 5A). However, with the combination of N146 deamidation plus NH_2_-terminal domain deletion, the αB N146D-NT mutant showed a 5 nm red shift with λ_max_ at 515 nm, with a decrease in fluorescence intensity similarly observed in the αB-NT mutant (Figure 5A). This suggested that, compared to WT, the NH_2_-terminal domain deletion alone resulted in a relatively relaxed structure with greater exposure of hydrophobic surfaces but decreased ANS binding intensity, whereas N-terminal domain deletion plus N146 deamidation exhibited increased surface exposure. Conversely, mutants with deletion of the COOH-terminal extension and COOH-terminal extension deletion plus N146 deamidation showed a blue shift in λ_max_ (i.e., λ_max_ at 496 nm and at 494 nm) and similar fluorescence intensity as WT (Figure 5B). This suggested that the COOH-terminal extension deletion reduced binding intensity of ANS and produced a relatively more compact structure, which became slightly more compact on N146 deamidation. Taken together, the results suggest that N146 deamidation and COOH-terminal extension deletion had greater effect on surface hydrophobicity compared to WT, causing larger shifts in fluorescence peaks, whereas deamidation alone caused an increase in binding intensity, not seen in any other mutant. These shifts in fluorescence peaks and changes in intensity suggest alterations in the microenvironments surrounding hydrophobic residues, and in turn changes in the surface hydrophobicity and tertiary structures of these mutants compared to WT αB-crystallin.

#### Determination of molecular mass by dynamic light scattering

To determine whether WT αB-crystallin and its mutants were able to oligomerize and form homomers, their molecular masses were determined using HPLC-coupled multi-angle light scattering (MALS) analysis (Wyatt Technology; Table 4). While WT αB-crystallin displayed a mass of 5.8×10^5^, the deamidated species (αB N146D) showed an increased mass of 3.1×10^6^. Upon deletion of the NH_2_-terminal domain (αB-NT) and NH_2_-terminal domain deletion plus deamidation (αB N146D-NT), increased masses of 3.0×10^6^ and 5.4×10^6^, respectively, were observed. Likewise, COOH-terminal extension deletion (αB-CT) and COOH-terminal extension deletion plus deamidation (αB N146D-CT) showed relative increase in mass of 1.2×10^6^ and 1.5×10^6^, respectively. Taken together, all mutants formed larger oligomers compared to WT αB-crystallin, and decreased in mass in the following order: αB N146D-NT > αB N146D > αB-NT > αB N146D-CT > αB-CT > WT αB-crystallin. These results also suggest that the NH_2_-terminally deleted mutants form larger oligomers than the COOH-terminally deleted mutants. In both cases, however, presence of the N146 deamidation produced greater increases in oligomer size than the NH_2_- or COOH-terminally deleted mutants alone.

**Table 4:** Molar mass determination of WT αB-crystallin and its mutants using the dynamic light scattering method (MALS).

**Crystallin species**	**Molecular mass (Da)**
WT αB	5.8×10^5^
αB N146D	3.1×10^6^
αB-NT	3.0×10^6^
αB N146D-NT	5.4×10^6^
αB-CT	1.2×10^6^
αB N146D-CT	1.5×10^6^

#### Chaperone activity of WT αB-crystallin and Its mutants

**Figure 6:**
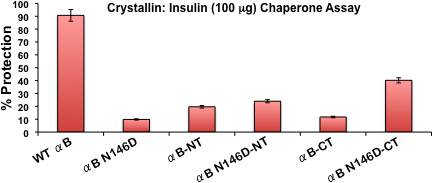
Comparison of chaperone activity of WT αB-crystallin and its mutant proteins. The chaperone activity, calculated as % protection, was assayed by measuring DTT-induced insulin (100 μg) aggregation in the presence of a chaperone/insulin ration (1:1) at 25 °C. Error bars=Percent Error (±1%).

To determine if the above observed structural changes also alter the functional property of the αB-crystallin mutants relative to the WT protein, chaperone activities were determined using insulin (100 μg) as the target protein in a 1:1 ratio (insulin: αB-crystallin protein), as previously described [24]. Light scattering caused by DTT-induced aggregation of insulin B chain was measured at 360 nm in the presence and absence of chaperone proteins, and the activity was represented as the percent protection against aggregation provided by the crystallin species (Figure 6). WT αB-crystallin provided about 90% protection against DTT-induced insulin aggregation. N146 deamidation of αB-crystallin reduced the protection to about 10%, as previously reported [24]. Upon NH_2_-terminal domain deletion, αB-NT provided about 20% protection, whereas upon COOH-terminal extension deletion in αB-CT only about 12% protection was observed. However, with the combination of deamidation plus NH_2_- or COOH-terminal deletion, chaperone activity was about 24% in αB N146D-NT and about 40% in αB N146D-CT. Chaperone activity across all αB-crystallin species decreased in the following order: WT αB-crystallin > αB N146D-CT > αB N146D-NT > αB-NT > αB-CT > αB N146D. These results suggest that deamidation alone greatly diminishes chaperone function. However, the COOH-terminal deletion alone is relatively more harmful to chaperone function than NH_2_-terminal deletion. Addition of N146 deamidation along with COOH-terminal extension deletion is relatively less deleterious compared to a combination of N146 deamidation and NH_2_-terminal domain deletion.

## Discussion

Although studies have identified deamidation [11,16-24,31] and truncation of crystallins [37-44] as the most abundant PTMs in aging and cataractous human lenses, the relative effects of these two PTMs, either individually or in combination, on structural and functional properties of the crystallins are not yet fully determined. Our recent report showed that deamidation alone had greater affect on the chaperone activity of αA-crystallin than deletion of the NH_2_-terminal domain or COOH-terminal extension [31]. This study also showed that although the N123 residue of αA-crystallin plays a crucial role in maintaining its chaperone function, both the NH_2_-terminal domain and COOH-terminal extension are equally important for chaperone activity. The conclusion was based on results showing that the chaperone activity lost by the αA-N123D mutant was partially or fully recovered following either of the above two deletions. In the present study we have taken a similar approach to determine the changes to the structural and functional properties of N146 deamidated and/or NH_2_-terminal domain or COOH-terminal extension deleted mutants relative to WT αB-crystallin. αB-crystallin contains two deamidation sites at N78 and N146, and we chose to focus on N146 in the present study because our previous work has shown that, among the two, N146D had a more profound effect on the chaperone activity of αB-crystallin [24].

In this study we have used His-tagged WT αB-crystallin and its NH_2_-terminal domain or COOH-terminal extension deleted and/or deamidated mutants. Several past studies have used His-tagged crystallins in their biophysical characterizations and chaperone activity assays, and we believe that the His-tag had minimal effect on the solubility of crystallin mutants. These previously published reports included His-tagged αA- [45], αB- [46], βB1- [45], and βB2-crystallin [47].

The major findings of the comparative studies of WT αB-crystallin and its mutant proteins are as follows: (1) The homomers of the COOH-terminal extension (residues no. 151–175) deleted αB (αB-CT) and its deamidated form (αB N146D-CT) became water insoluble (i.e., recovered in the inclusion bodies), whereas the deamidated αB-crystallin (αB N146D), NH_2_-terminal domain (residues no. 1–66) deleted αB-crystallin (αB-NT), and deamidated plus NH_2_-terminal deleted (αB N146D-NT) species remained water soluble. (2) Relative to a molecular mass of 5.8×10^5^ Da of WT αB-crystallin, all the αB mutants showed even higher molecular mass ranging from 1.2×10^6^ Da to 5.4×10^6^ Da. This suggested that, like WT αB-crystallin, all mutants also formed oligomers even after the above-described deamidation, deletion of either NH_2_-terminal domain or COOH-terminal extension, or combination of the two PTMs. (3) Chaperone activity across all αB-crystallin species decreased in the following order: WT αB > αB N146D-CT > αB N146D-NT > αB-NT > αB-CT > αB N146D. Specifically, relative to WT αB-crystallin with 90% protection against DTT-induced aggregation of insulin, substantial losses in chaperone activity (only 10% to 20% protection) were seen in αB N146D, NH_2_-terminal domain-deleted or COOH-terminal extension-deleted mutants. However, in the species with the combination of deamidation plus NH_2_- or COOH-terminal deletion, the percent protection was about 24% in αB N146D-NT and about 40% in αB N146D-CT. These results suggested the critical importance of the non-deamidated N146 residue, NH_2_-terminal domain and COOH-terminal extension in maintenance of chaperone activity of αB-crystallin, as was also suggested in our previous work [24]. Additionally, the above results suggested that the NH_2_-terminal domain is relatively more important than the COOH-terminal extension for the chaperone function of αB-crystallin. (4) CD spectroscopic studies showed that, relative to WT αB-crystallin, the mutants with deletion of the above-described NH_2_- and COOH-regions or deamidation showed increased β-sheet and decreased α-helical content, with the exception of αB N146D-CT, which showed a substantial increase in α-helix and decrease in β-sheet content. (5) Results of intrinsic Trp fluorescence suggested little change in Trp microenvironment of αB N146D relative to WT αB, but substantial alterations on deletion of COOH-terminal extension or a combination of this deletion plus deamidation. Because both Trp residues at positions 9 and 60 were lost on deletion of the NH_2_-terminal domain (residues no. 1–66), the total fluorescence spectra of the NH_2_-terminal domain-deleted mutants were recorded with excitation at 290 nm and emission spectra between 300 to 400 nm instead of intrinsic Trp fluorescence. A substantial decrease in fluorescence at 344 nm in αB-NT and at 339 nm in αB N146D-NT suggested major changes in microenvironment due to altered absorption of their aromatic amino acids. (6) The results of ANS binding studies to probe hydrophobic patches showed that relative to the WT αB-crystallin structure, the N146 deamidation, COOH-terminal extension deletion or a combination of this deamidation and deletion resulted in a relatively compact structure (a blue shift), whereas the NH_2_-terminal domain deletion and a combination of this deletion plus deamidation showed a relaxed structure (a red shift).

In our previous studies, deamidation alone of αA- and αB-crystallins [23,24] or deamidation plus COOH-terminal truncation in αA-crystallin [31], resulted in oligomers of higher molecular mass than those of WT αA- or WT αB-crystallins. Because these previous findings were similar to those described in the present study, it is believed that the higher molecular mass of mutants of αB-crystallin are of their oligomers rather than a result of aggregation of unfolded species.

On comparison of the above structural and functional changes of αB-crystallin mutants with our similar earlier study of αA-crystallin mutants relative to their WT proteins [23,31], several notable similarities were observed. For instance, like the above findings of αB-crystallin, the previous study showed insolubilization of αA-crystallin on deletion of the COOH-terminal extension, a substantially reduced chaperone activity on deamidation of αA-N123 to D, increased β-sheet content on deletion of NH_2_-terminal domain or COOH-terminal extension, and a slight red shift in the intrinsic Trp fluorescence on deletion of COOH-terminal extension. However, certain differences between the two studies were also observed, which included an opposite effect on the ANS binding to the two crystallin species, as seen by a blue shift in αA-crystallin in contrast to red shift in αB-crystallin upon NH_2_-terminal domain deletion. These differences were expected because, although αA- and αB-crystallins originated via gene duplication and have 57% sequence homology [48], our results show that individually isolated proteins containing either the NH_2_-terminal domain, core region or COOH-terminal extension of both αA- and αB-crystallins differed in their structural and functional properties [49]. These differences in the two crystallins were also noted in previous studies. Some such examples are: recombinant αA- and αB-crystallins differ in their secondary and tertiary structures, and relative to αA-crystallin, αB-crystallin showed a greater hydrophobicity and 4× more chaperone activity [50]; αA-crystallin was more stable to gamma irradiation relative to αB-crystallin [51]; and although αA- and αB-crystallins can each form oligomers independently, together or with other crystallins, their interactions with each other were 3× greater than their interactions with βB2- and γC-crystallins [52].

The crystal structures of bovine αA-crystallin (missing 59 NH_2_-terminal residues and 10 COOH-terminal residues, named αA_59–163_) and human αB-crystallin (missing 68 NH_2_-terminal residues and 13 COOH-terminal residues, named αA_68–162_) were recently published [53]. The bovine αA- and human αB-crystallin mutants retained their chaperone activity, polydispersity, and structural properties. In contrast to the mutants used in that study, we generated and studied the properties of human αB-crystallin mutants with NH_2_-terminal domain deletion (deletion of residues no. 1–66; αB-NT), deamidated plus NH_2_-terminal domain deletion (αB N146D-NT), COOH-terminal extension deletion (deletion of residues no. 151–175; αB-CT), and deamidated plus COOH-terminal extension deletion (αB N146D-CT). As shown in our study, although all mutants of αB-crystallin formed oligomers even after deamidation, deletion of either the NH_2_-terminal domain or COOH-terminal extension or a combination of these deletions and deamidation, their structural properties were substantially altered. Since the CD data of the NH_2_-terminal domain deleted αB-crystallin species with or without deamidation were analyzed using an earlier version of the SELCON software, the relative distribution of alpha-, beta- and random structures were quite different than the ones reported in our manuscript. The present data reported are those that were recovered using a newer version of the same software (SELCON3). Also, it is possible that after truncation these crystallin species may have attained more of a random structure.

The results of the crystal structure study [53] suggest that the COOH-terminal regions of both the crystallins were important for oligomerization and polydispersity. However, that same study also showed that with alcohol dehydrogenases as a target protein, an αB-crystallin mutant (αB_68–157_) with truncation of 68 NH_2_-terminal residues and 18 COOH-terminal residues exhibited a complete loss of chaperone activity whereas the above described human αB-crystallin mutant (αB_68–162_) showed the same level of chaperone activity as the WT αB-crystallin. However, in spite of the loss of chaperone activity, the αB_68–157_ mutant could still form large polydisperse oligomers, suggesting that the COOH-terminal residues 158–162 were needed for chaperone activity. As stated above, because of differences in deletion regions in αB-crystallin, our results and the results of the above-published report could not be compared in terms of structural changes. However, our results suggest that the NH_2_-terminal domain of αB-crystallin had a greater role in the chaperone activity compared to its COOH-terminal extension.

Several studies have shown in vivo deamidation of α-, β-, and γ-crystallins [10,22,54-56]. We have identified deamidation of N146 in a fragment of human αB-crystallin isolated from normal and cataractous lenses of 60- to 80-year-old donors [22]. Such deamidation of N146 has also been reported in bovine αB-crystallin [57], but its effect on the structural and functional properties of the crystallin was unknown until our present study, which describes such effects for the first time.

Like other sHSPs, α-crystallin also contains a highly conserved sequence of 80 to 100 residues (residues 64–142 in αA-crystallin and 67–146 in αB-crystallin) called the α-crystallin domain [5,58]. Based on similarities with the structure of other HSPs, it is believed that the NH_2_-terminal region (residue 1–63 in αA-crystallin and 1–66 in αB-crystallin) of α-crystallin forms an independently folded domain, whereas the COOH-terminal extension (residues 143–173 in αA- and 147–175 in αB-crystallin) is flexible and unstructured [5]. As stated above, the deletion of 41–58 residues from the NH_2_-terminal and 155–165 residues from the COOH-terminal in αB-crystallin resulted in altered complex assembly and chaperone activity [15]. The loss of the above NH_2_-terminal region resulted in a larger complex and greater polydispersity in αB-crystallin, but no loss in chaperone function.

The deamidated N146 exists within the conserved α-crystallin domain (residues 67–146) of αB-crystallin. Several past studies have demonstrated altered structural and functions properties of αA- and αB-crystallins following mutations in the NH_2_-terminal domain, α-crystallin domain or COOH-terminal extension. The α-crystallin domain is believed to engage in subunit–subunit interactions, because recombinant αB-crystallin containing only the α-crystallin core domain formed a dimer [59]. Also, two disease-related point mutations of a highly conserved Arg at equivalent positions in αA- (R116C) and αB-crystallin (R120G), located within the α-crystallin domain, caused structural changes that lead to hereditary cataracts [26,27,60,61]. Deletion of the last 17 amino acids of the COOH-terminal extension from human αB-crystallin caused precipitation, with reduced chaperone activity [62], and similarly a deletion of 25 residues from the COOH-terminus in *Xenopus* Hsp30C reduces its solubility and impairs chaperone activity [63]. On removal of NH_2_-terminal residues (partial or 1–56 residues of the NH_2_-terminus) and the COOH-terminal extension (partial or 32–34 residues of the COOH-terminus) of αA- and αB-crystallins, the proteins showed improper folding, altered chaperone activity, and formation of trimers or tetramers [13,64-66]. Residues 42–57 and residues 60–71 (located either in or near the NH_2_-terminal domain) of αB-crystallin interact with αA-crystallin [67,68]. Pin-array analysis has further shown that five peptide sequences of αB-crystallin (i.e., residues 37–54 [in the NH_2_-terminal domain], residues 75–82, 131–138, 141–148 [form β-strands in the conserved α-crystallin domain], and residues 155–166 [in the COOH-terminal extension]) interact with αA-crystallin [69]. Together these studies suggested that the NH_2_-terminal domain, α-crystallin domain and COOH-terminal extension of αB-crystallin are not only important for chaperone activity but also for interaction and oligomerization with αA-crystallin. Although the interaction sites in αA- and αB-crystallin homomers have been recently identified [53], the individual amino acids in the αA- and αB-crystallin subunits that interact with target proteins during chaperone activity have yet to be fully identified.

In conclusion, our study identified several structural changes in αB-crystallin following deamidation at N146D, deletion of NH_2_-terminal domain, deletion of COOH-terminal extension or a combination of the deamidation and either of the two deletions, and correlated these structural changes to their effect on the chaperone activity of the crystallin. Further, deamidation may serve as a signal for proteolysis as has been suggested by past studies [29,70]. Whether this signal is used during age- and cataract-related truncations of αA- and αB-, and other crystallins remains to be determined. We are presently attempting to find answers to these questions. In addition, our future studies will be focused on whether these post-translational modifications lead to cataract development in transgenic animals.

## References

[1] Bloemendal H, de Jong WW, Jaenicke R, Lubsen NH, Slingsby C, Tardieu A (2004). Ageing and vision: structure, stability and function of lens crystallins.. Prog Biophys Mol Biol.

[2] Andley UP (2007). Crystallins in the eye: function and pathology.. Prog Retin Eye Res.

[3] Klemenz R, Fröholi E, Steiger RH, Schäfer R, Aoyama A (1991). αB-Crystallin is a small heat shock protein.. Proc Natl Acad Sci USA.

[4] Bhat SP, Nagineni CN (1989). αB subunit of lens-specific protein α-crystallin is present in other ocular and non-ocular tissues.. Biochem Biophys Res Commun.

[5] de Jong WW, Caspers GJ, Leunissen JA (1998). Genealogy of the alpha-crystallin-small heat-shock protein superfamily.. Int J Biol Macromol.

[6] Renkawek K, Vooter CE, Bosman GJ, van Workum FP, de Jong WW (1994). Expression of alpha B-crystallin in Alzheimer’s disease.. Acta Neuropathol.

[7] Renkawek K, Stege GJ, Bosman GJ (1999). Dementia, gliosis and expression of the small heat shock proteins hsp27 and alpha B-crystallin in Parkinson’s disease.. Neuroreport.

[8] Horwitz J (1992). α-Crystallin can function as a molecular chaperone.. Proc Natl Acad Sci USA.

[9] McHaourab HS, Godar JA, Stewart PL (2009). Structure and mechanism of protein stability sensors: chaperone activity of small heat shock proteins.. Biochemistry.

[10] Hanson SRA, Hasan A, Smith DL, Smith JB (2000). The major in vivo modifications of the human water-insoluble lens crystallins are disulfide bonds, deamidation, methionine oxidation and backbone cleavage.. Exp Eye Res.

[11] Lund AL, Smith JB, Smith DL (1996). Modifications of the water-insoluble human lens α-crystallins.. Exp Eye Res.

[12] Groenen PJ, Merk KB, de Jong WW, Bloemendal H (1994). Structure and modifications of the junior chaperone α-crystallin. From lens transparency to molecular pathology.. Eur J Biochem.

[13] Bova MP, Mchaourab HS, Han Y, Fung BK (2000). Subunit exchange of small heat shock proteins. Analysis of oligomer formation of αA-crystallin and Hsp27 by fluorescence resonance energy transfer and site-directed truncations.. J Biol Chem.

[14] Pasta SY, Raman B, Ramakrishna T, Rao CM (2002). Role of C-terminal extensions of α-crystallins. Swapping the C-terminal extension of αA-crystallin to αB-crystallin results in enhanced chaperone activity.. J Biol Chem.

[15] Ghosh JG, Shenoy AK, Clark JI (2006). N- and C-terminal motifs in human αB-crystallin play an important role in the recognition, selection, and solubilization of substrates.. Biochemistry.

[16] Lapko VN, Purkiss AG, Smith DL, Smith JB (2002). Deamidation in human γS-crystallin from cataractous lenses is influenced by surface exposure.. Biochemistry.

[17] Harms MJ, Wilmarth PA, Kapfer DM, Steel EA, David LL, Bächinger HP, Lampi KJ (2004). Laser light-scattering evidence for an altered association of βB1-crystllin deamidated in the connecting peptide.. Protein Sci.

[18] Wilmarth PA, Tanner S, Dasari S, Nagalla SR, Rifiere MA, Bafna V, Pevzner PA, David LL (2006). Age-related changes in human crystallins determined from comparative analysis of post-translational modifications in young and aged lens: does deamidation contribute to crystallin insolubility?. J Proteome Res.

[19] Lampi KJ, Amyx KK, Ahmann P, Steel EA (2006). Deamidation in human lens βB2-crystallin destabilizes the dimmer.. Biochemistry.

[20] Hains PG, Truscott RJW (2007). Post-translational modifications in the nuclear region of young, aged, and cataract human lenses.. J Proteome Res.

[21] Hains PG, Truscott RJW (2010). Age-dependent deamidation of life-long proteins in the human lens.. Invest Ophthalmol Vis Sci.

[22] Srivastava OP, Srivastava K (2003). Existence of deamidated αB-crystallin fragments in normal and cataractous human lenses.. Mol Vis.

[23] Gupta R, Srivastava OP (2004). Deamidation affects structural and functional properties of human αA-crystallin and its oligomerization with αB-crystallin.. J Biol Chem.

[24] Gupta R, Srivastava OP (2004). Effect of deamidation of asparagine 146 on functional and structural properties of human lens αB-crystallin.. Invest Ophthalmol Vis Sci.

[25] Plater ML, Goode D, Crabbe MJ (1996). Effects of site-directed mutations on the chaperone-like activity of αB-crystallin.. J Biol Chem.

[26] Kumar LV, Ramakrishna T, Rao CM (1999). Structural and functional consequences of the mutation of a conserved arginine residue in αA and αB crystallins.. J Biol Chem.

[27] Liu Y, Zhang X, Luo L, Wu M, Zeng R, Cheng G, Hu B, Liu B, Liang JJ, Shang F (2006). A novel αB-crystallin mutation associated with autosomal dominant congenital lamellar cataract.. Invest Ophthalmol Vis Sci.

[28] Robinson NE, Robinson AB (2001). Molecular clocks.. Proc Natl Acad Sci USA.

[29] Robinson NE (2002). Protein deamidation.. Proc Natl Acad Sci USA.

[30] Selcen D, Engel AG (2003). Myofibrillar myopathy caused by novel dominant negative αB-crystallin mutations.. Ann Neurol.

[31] Chaves JM, Srivastava K, Gupta R, Srivastava OP (2008). Structural and functional roles of deamidation and/or truncation of N- or C-termini in human αA-crystallin.. Biochemistry.

[32] Laemmli UK (1970). Cleavage of structural proteins during the assembly of the head of bacteriophage T4.. Nature.

[33] Reddy MA, Bateman OA, Chakarova C, Ferris J, Berry V, Lomas E, Sarra R, Smith MA, Moore AT, Bhattacharya SS, Slingsby C (2004). Characterization of the G91del CRYBA1/3-crystallin protein: a cause of human inherited cataract.. Hum Mol Genet.

[34] Reddy GB, Kumar PA, Kumar MS (2006). Chaperone-like activity and hydrophobicity of α-crystallin.. IUBMB Life.

[35] Kundu B, Shukla A, Chaba R, Guptasarma P (2004). The excised heat-shock domain of αB-crystallin is folded, proteolytically susceptible trimer with significant surface hydrophobicity and a tendency to self-aggregate upon heating.. Protein Expr Purif.

[36] Kumar MS, Kapoor M, Sinha S, Reddy GB (2005). Insights into hydrophobicity and the chaperone-like function of αA- and αB-crystallins.. J Biol Chem.

[37] Srivastava OP (1988). Age-related increase in concentration and aggregation of degraded polypeptide in human lenses.. Exp Eye Res.

[38] Srivastava OP, Srivastava K, Harrington V (1999). Age-related degradation of βA3/A1-crystallin in human lenses.. Biochem Biophys Res Commun.

[39] Srivastava OP, Srivastava K (2003). βB2-crystallin undergoes extensive truncation during aging in human lenses.. Biochem Biophys Res Commun.

[40] Harrington V, McCall S, Huynh S, Srivastava K, Srivastava OP (2004). Crystallins in water soluble-high molecular weight protein fractions and water insoluble protein fractions in aging and cataractous human lenses.. Mol Vis.

[41] Harrington V, Srivastava OP, Kirk M (2007). Proteomic analysis of water insoluble proteins from normal and cataractous human lenses.. Mol Vis.

[42] Srivastava OP, Srivastava K (1998). Degradation of γD- and γS-crystallins in human lenses.. Biochem Biophys Res Commun.

[43] Srivastava OP, Srivastava K, Silney C (1996). Levels of crystallin fragments and identification of their origin in water soluble high molecular weight (HMW) proteins of human lenses.. Curr Eye Res.

[44] Srivastava K, Chaves JM, Srivastava OP, Kirk M (2008). Multi-crystallin complexes exist in the water-soluble high molecular weight protein fractions of aging normal and cataractous human lenses.. Exp Eye Res.

[45] Zhao Y, Ju F, Zhao Y, Wang L, Sun Z, Liu M, Gao L (2011). The expression of αA- and βB1-crystallin during normal development and regeneration, and proteomic analysis for the regenerating lens in *Xenopus laevis.*. Mol Vis.

[46] Hou YL, Hou WR, Ren ZL, Hao YZ, Zhang T (2008). cDNA, genomic sequence and overexpression of crystallin alpha-B Gene (*CRYAB*) of the Giant Panda.. Int J Biol Sci.

[47] Liu BF, Liang JJ (2005). Interaction and biophysical properties of human lens Q155* βB2-crystallin mutant.. Mol Vis.

[48] Horwitz J (2003). Alpha-crystallin.. Exp Eye Res.

[49] Asomugha CO, Gupta R, Srivastava OP (2011). Structural and functional properties of NH_2_-terminal domain, core domain, and COOH-terminal extension of αA- and αB-crystallins.. Mol Vis.

[50] Sun T-X, Das BK, Liang JJ-N (1997). Conformational and functional differences between recombinant human lens αA- and αB-crystallin.. J Biol Chem.

[51] Fujii N, Nakamura T, Sadakane Y, Saito T, Fujii N (2007). Differential susceptibility of alpha A- and alpha B-crystallin to gamma-ray irradiation.. Biochim Biophys Acta.

[52] Fu L, Liang JJ-N (2002). Detection of protein-protein interactions among lens crystallins in a mammalian two-hybrid system assay.. J Biol Chem.

[53] Laganowsky A, Benesch JLP, Landau M, Ding L, Sawaya MR, Cascio D, Huang Q, Robinson CV, Horwitz J, Eisenberg D (2010). Crystal structures of truncated alphaA and alphaB crystallins reveal structural mechanisms of polydispersity important for eye lens function.. Protein Sci.

[54] Takemoto L (1999). Increased deamidation of asparagine-101 from alpha-A crystallin in the high molecular weight aggregate of the normal human lens.. Exp Eye Res.

[55] Lampi KJ, Kim YH, Bächinger HP, Boswell BA, Lindner RA, Carver JA, Shearer TR, David LL, Kapfer DM (2002). Decreased heat stability and increased chaperone requirement of modified human βB1-crystallins.. Mol Vis.

[56] Lampi KJ, Oxford JT, Bächinger HP, Shearer TR, David LL, Kapfer DM (2001). Deamidation of human βB1 alters the elongated structure of the dimer.. Exp Eye Res.

[57] Groenen PJ, van Dongen M, Voorter CE, Bloemendal H, de Jong WW (1993). Age-dependent deamidation of αB-crystallin.. FEBS Lett.

[58] Caspers GJ, Leunissen JA, de Jong WW (1995). The expanding small heat-shock protein family, and structure predictions of the conserved “alpha-crystallin domain”.. J Mol Evol.

[59] Feil IK, Malfois M, Hendle J, van Der Zandt H, Svergun DI (2001). A novel quaternary structure of the dimeric α-crystallin domain with chaperone-like activity.. J Biol Chem.

[60] Bova MP, Yaron O, Huang Q, Ding L, Haley DA, Stewart PL, Horwitz J (1999). Mutation R120G in αB-crystallin, which is linked to a desmin-related myopathy, results in an irregular structure and defective chaperone-like function.. Proc Natl Acad Sci USA.

[61] Cobb BA, Petrash JM (2000). Structural and functional changes in the αA-crystallin R116C mutant in hereditary cataracts.. Biochemistry.

[62] Smulders RHPH, Carver JA, Lindner RA, van Boekel MA, Bloemendal H, de Jong WW (1996). Immobilization of the C-terminal extension of bovine αA-crystallin reduces chaperone-like activity.. J Biol Chem.

[63] Fernando P, Heikkila JJ (2000). Functional characterization of Xenopus small heat shock protein, Hsp30C: the carboxyl end is required for stability and chaperone activity.. Cell Stress Chaperones.

[64] Takemoto L, Emmons T, Horwitz J (1993). The C-terminal region of alpha-crystallin: involvement in protection against heat-induced denaturation.. Biochem J.

[65] Aziz A, Santhoshkumar P, Sharma KK, Abraham EC (2007). Cleavage of the C-terminal serine of human αA-crystallin produces αA_1–172_ with increased chaperone activity and oligomeric size.. Biochemistry.

[66] Andley UP, Shashank M, Griest TA, Petrash JM (1996). Cloning, expression, and chaperone-like activity of human αA-crystallin.. J Biol Chem.

[67] Sreelakshmi Y, Santhoshkumar P, Bhattacharyya J, Sharma KK (2004). αA-crystallin interacting regions in the small heat shock protein, αB-crystallin.. Biochemistry.

[68] Sreelakshmi Y, Sharma KK (2006). The interaction between αA- and αB-crystallin is sequence-specific.. Mol Vis.

[69] Ghosh JG, Clark JI (2005). Insights into the domains required for dimerization and assembly of human αB-crystallin.. Protein Sci.

[70] Inaba M, Gupta KC, Kuwabara M, Takahashi T, Benz EJ, Maede Y (1992). Deamidation of human erythrocyte protein 4.1: possible role in aging.. Blood.

